# Flocculation-Related Gene Identification by Whole-Genome Sequencing of Thauera aminoaromatica MZ1T Floc-Defective Mutants

**DOI:** 10.1128/AEM.02917-15

**Published:** 2016-03-07

**Authors:** P. Prombutara, M. S. Allen

**Affiliations:** a Department of Biological Sciences, University of North Texas, Denton, Texas, USA; b Center for Biosafety and Biosecurity, University of North Texas Health Science Center, Fort Worth, Texas, USA; c Department of Molecular and Medical Genetics, University of North Texas Health Science Center, Fort Worth, Texas, USA; Wageningen University

## Abstract

Thauera aminoaromatica MZ1T, a floc-forming bacterium isolated from an industrial activated-sludge wastewater treatment plant, overproduces exopolysaccharide (EPS), leading to viscous bulking. This phenomenon results in poor sludge settling and dewatering during the clarification process. To identify genes responsible for bacterial flocculation, a whole-genome phenotypic-sequencing technique was applied. Genomic DNA of MZ1T flocculation-deficient mutants was subjected to massively parallel sequencing. The resultant high-quality reads were assembled and compared to the reference genome of the wild type (WT). We identified nine nonsynonymous mutations and one nonsense mutation putatively involved in EPS biosynthesis. Complementation of the nonsense mutation located in an EPS deacetylase gene restored the flocculating phenotype. The Fourier transform infrared (FTIR) spectra of EPS isolated from the wild type showed a reduced C=O peak of the *N*-acetyl group at 1,665 cm^−1^ compared to the spectra of MZ1T floc-deficient mutant EPS, suggesting that the WT EPS was partially deacetylated. Gene expression analysis also demonstrated that the putative deacetylase gene transcript increased before flocculation occurred. These data suggest that targeting deacetylation processes via direct chemical modification of EPS or enzyme inhibition may prove useful in combating viscous bulking in this and related bacteria.

## INTRODUCTION

Viscous bulking occurring during the clarification process of activated sludge is responsible for poor sludge settling and dewatering, leading to high-biochemical-oxygen-demand (BOD) effluent and often failure of the whole wastewater treatment process. This phenomenon is caused by overgrowth of nonfilamentous floc-forming organisms, which produce high levels of exopolysaccharide (EPS). The sludge is generally negatively charged, and thus, cationic synthetic polymers are commonly added to neutralize the sludge surface charge, facilitating flocculation and settling. It is estimated that 25 to 50 million kg of polymers, costing $130 million, is utilized annually in the United States for the treatment of viscous bulking (1). Furthermore, the use of synthetic chemicals in wastewater treatment results in a polluted environment after incineration and disposal of the sludge.

Thauera aminoaromatica MZ1T was originally isolated from an industrial wastewater treatment plant experiencing a prolonged episode of viscous bulking. The EPS of this floc-forming bacterium was found to be comprised of four monosaccharides: rhamnose, galacturonic acid, *N*-acetylglucosamine, and *N*-acetyl fucosamine. The EPS composition of MZ1T was found to be similar to that of Zoogloea ramigera with respect to the presence of aminosugars. Moreover, Fourier transform infrared (FTIR) and nuclear magnetic resonance (NMR) spectroscopies uncovered the presence of possible nonsugar substitutes esterified to the exopolysaccharide of MZ1T (2). EPS modification was found to be involved in the development of biofilm and virulence in bacteria. Deacetylation of the poly-*N*-acetylglucosamine polymer in Staphylococcus epidermidis is necessary for polysaccharide attachment to the cell surface and the ability to form biofilm. S. epidermidis deacetylase-defective mutants also fail to protect the cells from major components of the innate immune response (3). However, deacetylation can be required for polysaccharide synthesis. For instance, mutation in the putative active site of the deacetylase gene, *pelA*, in Pseudomonas aeruginosa prevents polymerization of Pel polysaccharide (4).

Although little is known about the flocculation mechanism of MZ1T, two major mechanisms responsible for flocculation have been proposed. One is bridging by divalent cations, mainly Ca^2+^, of negatively charged functional groups of the side chain of EPS on the bacterial surface structures (5). Another potential mechanism of flocculation is binding of EPS to polysaccharide binding proteins (lectins) attached to the cell surface. The latter has been found to be the primary flocculation mechanism in the yeast Saccharomyces cerevisiae (6). Understanding the mechanisms of flocculation in MZ1T may ultimately enhance wastewater removal performance. The lessons learned here may also find applications in other biotechnological processes where cells must be removed by costly centrifugation or filtration postproduction.

Here, we employed a phenotype-sequencing approach in which mutations causing the phenotype can be identified directly from the sequencing of multiple independent mutant strains. In this study, flocculation mutants were created by chemical mutagenesis and screened for flocculation *in vitro*. The full genomes of two of these T. aminoaromatica strain MZ1T flocculation-deficient mutants were sequenced using the Ion Torrent Personal Genome Machine (PGM), an integrated semiconductor-based genome-scale DNA sequencer. The millions of short reads generated were mapped to the reference genome of wild-type (WT) T. aminoaromatica MZ1T. Among 75 mismatches identified, the EPS deacetylase gene (*tmz1t_3249*) contains a nonsense mutation, and we demonstrate that complementation with this gene in the T. aminoaromatica MZ1T 20A mutant can rescue the flocculation phenotype. We subsequently determined the temporal gene expression of the identified deacetylase gene and a putative EPS-biosynthetic gene in wild-type MZ1T from early-log-phase planktonic cultures through the postflocculation stationary phase, using droplet digital PCR of mRNA-derived cDNA.

## MATERIALS AND METHODS

### Bacterial strains, growth conditions, and media.

The T. aminoaromatica MZ1T wild-type strain was originally isolated from the wastewater treatment plant of the Eastman Chemical Company in Kingsport, TN (7). A rifampin-resistant (Rif^r^) mutant of MZ1T was isolated by repeated plating on Stoke's agar plates (1.5% Bacto agar in Stoke's broth [5 g polypeptone, 0.2 g MgSO_4_·7H_2_O, 0.15 g Fe NH_4_SO_4_), 0.1 g sodium citrate, 0.05 g CaCl_2_, 0.05 g MnSO_4_, 0.01 g FeCl_3_·6H_2_O]) amended with 100 μg/ml rifampin (Sigma) and was used for all studies. *T*. aminoaromatica MZ1T wild type and mutants defective in floc formation were routinely grown in Stoke's broth. The medium was sterilized by autoclaving, and the following filter-sterilized vitamin solutions were added to yield the indicated final concentrations: cyanocobalamin, 0.5 mg/liter; thiamine hydrochloride, 0.4 mg/liter; and biotin, 0.4 mg/liter. Otherwise, Thauera defined medium (TDM), modified from that used by Rabus and Widdel (8), was used for growing the MZ1T and mutant strains for EPS extraction and purification. All MZ1T strains were grown aerobically at 30°C in a shaking incubator at 225 rpm.

### Mutagenesis.

Chemical mutagenesis of rifampin-resistant MZ1T was carried out using *N*-methyl-*N*′-nitro-*N*-nitrosoguanidine (NTG) from Sigma Chemical Co. in a manner similar to that outlined by Coschigano et al. (9) and modified by Allen (10). Briefly, cells from frozen stock were grown in Stoke's broth for 48 h to mid-log phase, and 10 ml of MZ1T culture was centrifuged at 5,000 × *g* to pellet the cells. The cells were resuspended in 9.9 ml of 100 mM sodium citrate buffer (pH 5.5), and 0.1 ml of NTG stock solution in the same buffer was added to yield a final NTG concentration of 100 μg/ml. The tubes were incubated without shaking for 30 min. The cells were pelleted by centrifugation and washed three times with 100 mM potassium phosphate (pH 7.0). The treated cells were serially diluted and plated on Stoke's agar plates. The resultant colonies were picked and visually screened for the loss of floc formation capacity in 10-ml culture tubes containing 2 ml Stoke's broth.

### EPS extraction and purification.

The water-soluble EPS was extracted from 1 liter of the culture medium of the T. aminoaromatica MZ1T wild type and mutants after 7 days by removing cells using centrifugation (8,000 × *g* for 15 min) and then purified from the supernatant by tangential-flow filtration against deionized water using a Pellicon-2 Mini Holder equipped with a Biomax 100K filter (Millipore, USA). One hundred milliliters of the retentate containing the concentrated polysaccharide was diluted back to 1 liter, and the process was repeated a total of three times. The resulting desalted polysaccharide solution was then frozen at −80°C and lyophilized (2). The polysaccharide content was assayed by the phenol-H_2_SO_4_ method (11).

### FTIR analysis.

For FTIR measurements, 100 μl of the desalted crude EPS solution (10 mg/ml) was dried and clamped against the ATR crystal (germanium). The absorption spectrum between 400 and 4,000 cm^−1^ was measured by co-adding 100 scans and subtracting both the background and atmospheric water. Spectra were recorded using attenuated total reflectance (ATR) on a Bio-Rad FTS 6000 FTIR spectrometer.

### Deacetylation of EPS.

Deacetylation of EPS purified from T. aminoaromatica MZ1T mutants was performed as follows. Twenty milligrams of dried EPS was reacted with 10 ml of 45% NaOH for 15 min at 100°C. After cooling, absolute ethanol (1:1) was added to the mixture and pelleted by centrifugation at 10,000 × *g* for 10 min at 4°C. The sediment was dried and analyzed by FTIR spectrophotometry as described above.

### Ion library and template preparation.

Library preparation was performed using the NEBNext Fast DNA Fragmentation and Library Prep set for Ion Torrent (New England BioLabs), following the manufacturer's instructions. Briefly, 1 μg of 20A and 39A MZ1T mutants' genomic DNA was enzymatically fragmented and end repaired. Each library fragment was barcoded and ligated to P1 adapters. For optimal sequencing results, a DNA library with a mean size of 290 to 330 bp was purified and size selected using AMPure XP beads (Beckman Coulter). Product quality and quantity were confirmed and measured on an Agilent 2100 Bioanalyzer system (Agilent Technologies). Molar equivalents of both 20A and 39A MZ1T DNA libraries were calculated, pooled, and diluted to 8.3 nM (5 × 10^9^ molecules/μl). The sample was amplified by emulsion PCR using the Ion Torrent One Touch system, following the manufacturer's instructions. The resultant beads were subsequently purified and enriched on the Ion ES automated instrument.

### Ion sequencing.

The Ion sequencing kit was used for sequencing according to the manufacturer's instructions. First, the PGM sequencer was cleaned, initialized, and pH calibrated. Enriched template Ion sphere particles (ISPs) from the above-mentioned step were annealed to sequencing primers at 95°C for 2 min and at 37°C for 2 min in a thermal cycler. Subsequently, 3 μl of the sequencing polymerase was added, and the reaction mixture was incubated for 5 min at room temperature. While the enriched template ISPs were being prepared for sequencing, a new Ion v316 chip was tested on the PGM sequencer. Then, the Ion chip was loaded with the ISPs according to the manufacturer's protocol. Finally, the chip was loaded on the Ion PGM system and run. After completion (2 h), the Ion data were retrieved and processed. Reads were mapped to the T. aminoaromatica MZ1T reference genome retrieved from the NCBI database (accession number NC_011662.2) using NextGENe software (Softgenetics, State College, PA); single nucleotide polymorphisms (SNPs) and short insertion/deletions (indels) were called from the consensus sequence, and coverage scores were also calculated.

### SNP validation by Sanger sequencing.

A candidate gene with an SNP detected by Ion Torrent sequencing was PCR amplified from the corresponding mutant genome and cloned into the pCR2.1 plasmid using the TOPO TA cloning kit (Invitrogen). pCR 2.1 plasmids harboring the mutant candidate gene were transformed into Escherichia coli TOP10 cells (Invitrogen) using the manufacturer's heat shock procedure. Transformants were plated on LB agar plates containing 50 mg/ml kanamycin, which were overlaid with 40 μl of 40-mg/ml X-Gal (5-bromo-4-chloro-3-indolyl-d-galactopyranoside) to facilitate blue/white screening of colonies. Several of the white colonies generated were inoculated into 2 ml liquid LB medium containing kanamycin. Overnight cultures were pelleted by centrifugation at 10,000 × *g* for 1 min and purified with plasmid minipreps (5 Prime FastPlasmid minikit; Fisher Scientific). The resultant plasmids (150 ng/μl) were sent to Eurofins MWG Operon (Huntsville, AL) for Sanger sequencing.

### Mutant complementation.

Candidate genes ware amplified from the genomic DNA of the MZ1T wild type by PCR. PCR amplification of the full-length genes from the genomic DNA involved 30 cycles of PCR amplification with Q5 proofreading polymerase (NEB), followed by the addition of 2.5 U *Taq* polymerase and incubation at 72°C for 10 min to add 3′-A overhangs in order to facilitate cloning into the pCR2.1 TA cloning vector (Invitrogen). The cloned fragments were then transformed into E. coli TOP10 cells (Invitrogen) as previously described. The resultant plasmid was cut with XbaI and HindIII enzymes (NEB) in a double digestion at 37°C for 90 min. The broad-host-range plasmid pRK415 (12) was also digested in the same manner. The gene fragments purified by gel electrophoresis were ligated into the cut pRK415 vector using T4 DNA ligase (NEB) according to the manufacturer's instructions to yield pRK415::*tmz1t_*gene locus (where the specific gene locus is inserted in the gene name) (Table 1). Transformation of pRK415 into chemically competent E. coli TOP10 cells (Invitrogen) was achieved as previously described, and the resultant transformants were screened on LB agar plates containing 15 mg/ml tetracycline. Conjugation of the pRK415 expression vector and its derivatives into the appropriate MZ1T mutant strains was performed via triparental mating. Briefly, 10-ml cultures of E. coli harboring pRK415::*tmz1t*_gene locus (Table 1 shows the specific constructs) (donors), MZ1T mutants (recipients), and E. coli harboring pRK2013 (helper strain) were individually grown to an approximate optical density at 600 nm (OD_600_) of 0.5 in Stoke's broth with 100-μg/ml rifampin for MZ1T or LB with suitable antibiotics for E. coli strains. Cells were collected, and the supernatant fraction was decanted. The cell pellets were suspended in an equal volume of phosphate-buffered saline (PBS) (pH 7.0). This washing was repeated twice, and the third time, the donor, recipient, and helper strains were combined into a single tube and pelleted together by centrifugation at 8,000 × *g* for 1 min The supernatant fraction was decanted, and the pellet was suspended in 1 ml PBS. Aliquots containing 100 μl of cell suspension were applied to autoclave-sterilized 25-mm, 0.2-μm-pore-size filter disks on nonselective Stoke's agar plates and incubated at 30°C for 48 h. Following incubation, the filter disks were aseptically removed and transferred to minimal TDM containing 15 g/ml tetracycline and 100 μg/ml rifampin. Once the culture appeared turbid, aliquots containing 100 μl of cell suspension were spread plated onto Stoke's agar plates containing the same antibiotics as the TDM. These plates were then incubated for 48 h at 30°C and screened for the appearance of colonies. The complemented MZ1T colonies were distinguished from E. coli colonies using an oxidase test. Successful MZ1T transconjugants were screened for flocculation to ensure that the proper phenotype had been successfully rescued. All MZ1T strains were similarly transformed with the empty pRK415 as a negative control.

**TABLE 1:** Candidate genes involved in MZ1T flocculation^*a*^

% SNP	Gene	Gene product function	Type of mutation	Base change (NextGENe software)	Amino acid change	Coverage	Mutant strain
100	*tmz1t_3249*	Polysaccharide deacetylase	Nonsense	G to A	W111 stop	12×	20A
90	*tmz1t_3637*	Family 2 glycosyltransferase	Frameshift	G/−^*d*^	V162A^*b*^	15×	20A
93	*tmz1t_3801*	Polysaccharide biosynthesis CapD	Nonsynonymous	T to G	L102R^*c*^	29×	20A
100	*tmz1t_0834*	Type IV pilus assembly protein	Nonsynonymous	G to A	A36T^*b*^	17×	39A
100	*tmz1t_1376*	ABC transporter	Nonsynonymous	G to A	G170R^*c*^	19×	39A
70	*tmz1t_1383*	Von Willebrand factor	Nonsynonymous	G to A	V162A^*b*^	17×	39A
100	*tmz1t_1679*	PAS/PAC sensor containing diguanylate cyclase	Nonsynonymous	G to A	T615I^*b*^	14×	39A
100	*tmz1t_2095*	CzcA family heavy-metal efflux pump	Nonsynonymous	A to T	V57E^*c*^	17×	39A
90	*tmz1t_3143*	Diguanylate cyclase	Frameshift	C/−^*d*^	L508W^*b*^	11×	39A
100	*tmz1t_3810*	Glucose-1-phosphate thymidylyltransferase	Nonsynonymous	G to A	R128H^*b*^	22×	39A

a The table shows loci with %SNP values of >75% and coverage of >10×. The percentage of the single most prevalent nonreference base in the aligned column is also shown.

b Amino acid change predicted to result in mild property change.

c Amino acid change predicted to result in severe property change.

d Base deletion.

### ddPCR.

Extraction of total RNA was performed from 18-, 24-, 36-, 48-, 72-, and 96-h MZ1T wild-type cultures using a FastRNA pro kit (MP Bio) following the manufacturer's instructions. To remove contaminating genomic DNA, RNA samples were treated using Qiagen's RNeasy on-column DNase I (2.7 U DNase I, 10 μg RNA), followed by Qiagen RNeasy MinElute (for DNase I removal), according to the manufacturer's protocol, before proceeding with cDNA synthesis. The RNA concentration was determined using a Nanodrop ND-1000 instrument (Nanodrop Technologies, Wilmington, DE). cDNA was synthesized from the extracted RNA using the iScript cDNA synthesis kit (Bio-Rad). Approximately 10 μg of RNA was used for cDNA synthesis in a 20-μl volume. The samples were diluted with 10× RNase-free double-distilled H_2_O (ddH_2_O) and stored at −80°C for later use. Droplet digital PCR (ddPCR) mixtures containing 1 μl of cDNA sample; 2 μl of primers (100 nM) for MZ1T EPS biosynthesis (*tmz1t_*3244) (F′, CTCTTCAACAAGGGCGAGAG, and R′, CAGCGTGTAGACGACCATCA), EPS deacetylase (*tmz1t*_3249) (F′, AACGACTGGGAACGCAAGTA, and R′, CTGACCAGGAAGACCGTTG), or glyceraldehyde-3-phosphate dehydrogenase (GAPDH) gene transcript measurement (F′, AGGTCGTGCATGAAAACCTC, and R′, TTCAGCTCCGGGTAGATCAG); 10 μl of Bio-Rad ddPCR supermix; and 7 μl of ddH_2_O were assembled. A 20-μl aliquot was pipetted into each sample well of an eight-channel disposable droplet generator cartridge (Bio-Rad, Hercules, CA, USA). A 70-μl volume of Droplet Generation Oil for EvaGreen (Bio-Rad) was then loaded into each of the eight oil wells, and the cartridge was placed into the droplet generator (Bio-Rad). After droplet generation, 40 μl of the generated droplet emulsion was transferred to a new 96-well PCR plate (Eppendorf) and amplified in a C100 Thermal Cycler (Bio-Rad). The amplification conditions were 10 min of DNA polymerase activation at 95°C, followed by 40 cycles of a two-step thermal profile of 30 s at 94°C for denaturation and 60 s at 60°C for annealing and extension, followed by a final hold of 10 min at 98°C for droplet stabilization and cooling to 4°C. The temperature ramp rate was set to 2.5°C/s, and the lid was heated to 105°C, according to the Bio-Rad recommendations. After the thermal cycling, the plates were transferred to a droplet reader (QX-200; Bio-Rad). The software package provided with the ddPCR system (QuantaSoft 1.3.2.0; Bio-Rad) was used for data acquisition. Annealing temperatures were experimentally optimized (data not shown) using a temperature gradient of the thermal cycle to avoid nonspecific products and primer dimers. The gene transcript abundance was quantified in triplicate for each cDNA sample.

## RESULTS

### Mutagenesis of MZ1T and isolation of floc-defective mutants.

**FIG 1:**
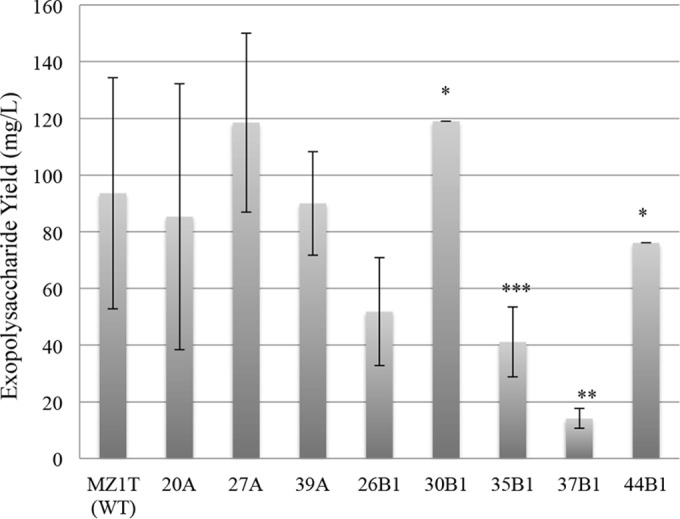
Average yields of EPS from floc^+^ and floc-defective strains. MZ1T EPS was recovered from 1-liter cultures of MZ1T WT and mutant strains. The yield values are averages of at least three replicates, except where noted. The error bars represent standard deviations. **, the mean value is significantly different from the mean of the wild type at a 95% confidence interval; ***, the mean is significantly different from that of the wild type at a confidence interval of 90%; *, value from a single experiment.

**FIG 2:**
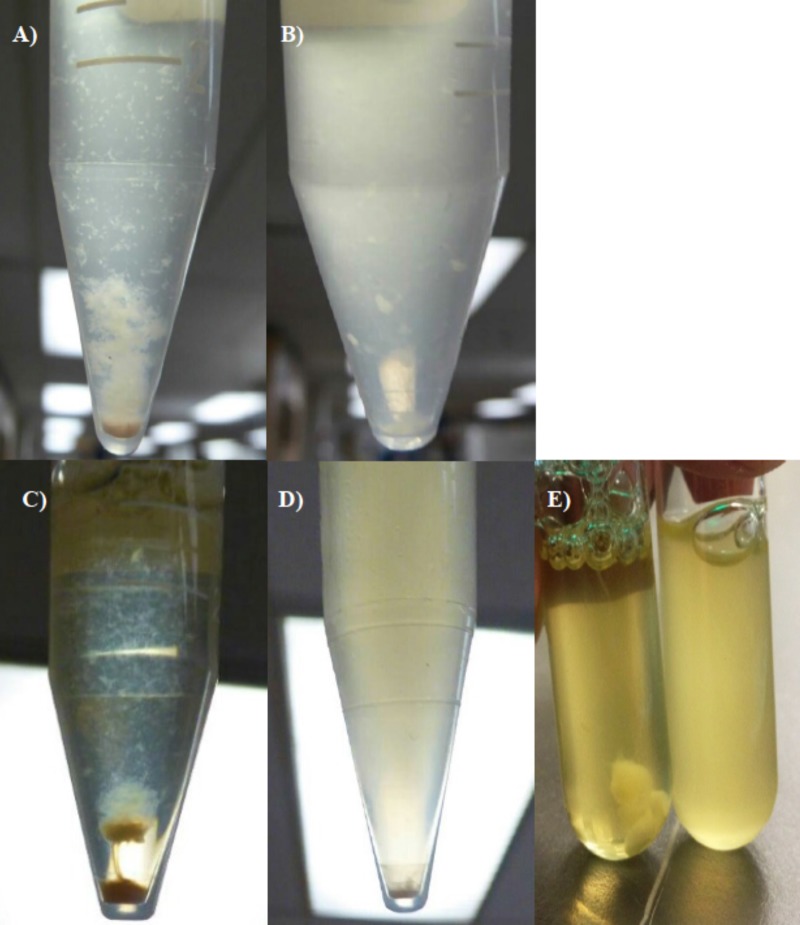
Complementation of MZ1T 20A by EPS deacetylase (*mz1t_3249*) and flocculation-like cell clumping of E. coli. (A) MZ1T WT. (B) MZ1T 39A. (C) MZ1T 20A carrying pRK415::*mz1t_3249*. (D) MZ1T 20A carrying empty plasmid pRK415. (E) Flocculation-like cell clumping of E. coli carrying pRK415::*mz1t_3249* (left tube) compared to E. coli carrying empty plasmid pRK415 (right tube).

Cultures of a spontaneous rifampin-resistant mutant were treated with NTG, and the resultant colonies were individually screened for loss of floc formation capacity in 10-ml test tubes containing 2 ml Stoke's broth. From approximately 2,000 colonies screened, eight putative floc-defective mutants were isolated. Their strain designations and EPS yields are shown in Fig. 1. Since floc formation is one of the few distinguishing characteristics of MZ1T, floc-defective mutants were verified by rRNA intergenic space analysis (RISA) and/or BOX PCR. Additionally, 16S rRNA genes were amplified, cloned, and sequenced from mutant isolates 20A, 26B, and 39A. Of the eight chemically derived floc-defective mutants originally isolated, mutants 39A and 44B1 were later found to form flocs to a greatly reduced degree and only after a period of 3 to 4 days compared with 2 days for the wild-type strain (Fig. 2A and B). Flocculation in these two mutants is limited to only a modest amount of cell aggregation, while the remainder of the culture broth remains turbid.

### EPS production in floc-defective mutants.

EPS quantities represent averages of separate EPS extractions from the supernatant fractions of three individual 1-liter cultures. EPS production ranged from wild-type levels to a significantly reduced yield, approximately 10% of wild type. In fact, mean yields from mutants 37B1 and 35B1 were found to be significantly different from the mean yield of the wild type at the 95% and 90% confidence interval, respectively.

### Glycosyl composition of EPS from floc-defective mutants.

The glycosyl composition of EPS isolated from each of the eight putative floc-defective mutants was determined using the alditol acetate and trimethylsilyl (TMS) methods, as previously described (10). Both methods revealed the presence of the same monosaccharides previously detected in EPS extracts from floc^+^ MZ1T (i.e., T. aminoaromatica MZ1T strains exhibiting wild-type-level floc formation).

### Genome sequencing of floc-defective mutants MZ1T 39A and 20A.

The Ion Torrent PGM semiconductor-sequencing platform was used to sequence the genomes of mutant MZ1T floc-defective strains 39A and 20A previously generated by chemical mutagenesis. High loading density (76%) of Ion Torrent chips (v316 chip) was accomplished and provided several hundred thousand (134-bp average length) reads. The total numbers of bases were 64.21 (39A) and 102.20 (20A) million, with average genomic coverage of 15- and 24-fold (15× and 24×), respectively (NCBI Sequence Read Archive accession numbers SRR2960577 and SRR2960208). The reads were compared to the T. aminoaromatica MZ1T reference genome, and variants were identified via analysis of the mapped reads. In addition, the SNPs were filtered based on SNP percentages (SNP%). SNPs with an SNP% of ≥75% (for example, number of SNPs, 3; read depth, 4) were chosen. The 75% cutoff for SNP selection was set by considering potential sequencing errors that can be generated by the massively parallel sequencing method. Subsequently, a total of 75 putative point mutations, insertions, and deletions were mapped out from the wild-type genome reference. Most of the SNPs showed that a strong bias occurred at GC sites (GC to AT), as would be expected for NTG-generated mutations. Interestingly, a mutation of *rhoB* was identified in both MZ1T mutants, caused by a transversion (T to A) leading to an amino acid substitution (glutamine is replaced by leucine). The encoded protein is the target of the antibiotic rifampin. Its identification was suggestive of a successful experimental design, since MZ1T 39A and 20A were derived from the same spontaneous Rif^r^ mutant of the wild type.

### SNP validation using PCR and Sanger sequencing.

We picked nonsynonymous and frameshift mutations in EPS biosynthesis, type IV pilus assembly, global regulatory, and EPS export-related genes detected by Ion Torrent to test and verify by Sanger sequencing. The Sanger-sequencing results confirmed these genes were altered in the mutant strains (Table 1). Among them, the EPS deacetylase gene of the MZ1T 20A mutant was confirmed to have a nonsense mutation introducing a stop codon in the middle of the gene. Subsequently, the wild-type versions of the verified candidate genes were subcloned into the broad-host-range plasmid pRK415 to further test complementation of the floc-defective MZ1T mutant phenotypes.

### Complementation.

pRK145 plasmids carrying wild-type candidate genes *tmz1t_3249*, *tmz1t _3637*, and *tmz1t _3801* were used to complement the MZ1T 20A mutant by triparental mating. Among these genes, complementation of the MZ1T 20A mutant strain with the pRK145 plasmid bearing an EPS deacetylase gene (*tmz1t_3249*) restored the flocculation phenotype, but no flocculation was observed in the MZ1T 20A mutant bearing plasmid pRK415 (Fig. 2C and D) without the cloned insert. Intriguingly, E. coli carrying pRK415::*mz1t_3249* formed clumping cells similar to the flocculation of WT MZ1T, in contrast to E. coli carrying the empty pRK415 vector (Fig. 2E). In addition, the MZ1T 39A mutant was also transformed with pRK415 plasmids that carry *tmz1t_0834*, *tmz1t _1376*, *tmz1t _3143*, and *tmz1t* _*3810* genes; however, none of these plasmids could restore flocculation to wild-type levels.

### FTIR characterization studies of EPS.

**FIG 3:**
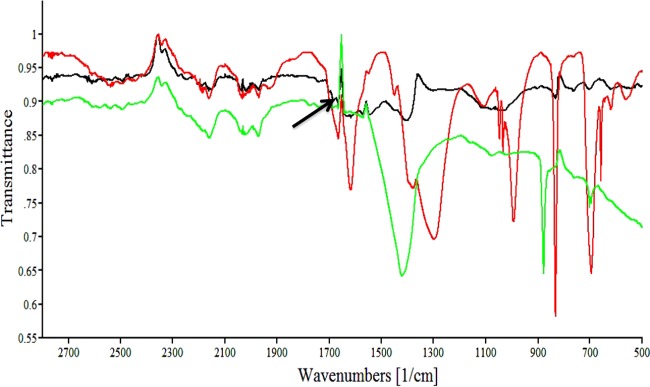
Comparison of FTIR spectra of chemically deacetylated MZ1T mutant 20A EPS (green line), MZ1T mutant 20A EPS (red line), and MZ1T wild-type EPS (black line). The arrow indicates the C=O stretching vibration peak of the *N*-acetyl group at 1,655 cm^−1^.

FTIR bands of purified EPSs from all MZ1T strains consistent with polysaccharide characteristic peaks were detected at 1,400 cm^−1^, corresponding to the symmetrical stretching C=O of COO^−^ carboxyl groups, and at 950 to 1,200 cm^−1^, the C—H stretching of alcohols, C—OH stretching, and C—O stretching of C—O—C. Moreover, purified EPSs from MZ1T 20A, which completely lost flocculation ability, demonstrated interesting altered peaks compared to the MZ1T WT at 1,665 cm^−1^, representing a reduced C=O stretching vibration peak of the *N*-acetyl group, whereas the N—H deformation vibration peak of a secondary amine group at 1,527.84 cm^−1^ increased compared to the spectra of purified MZ1T mutant EPS. Also, chemically deacetylated EPS of the MZ1T 20A mutant showed a diminished C=O stretching peak of the *N*-acetyl group, similar to the IR pattern of MZ1T wild-type EPS (Fig. 3).

### Expression of genes involved in MZ1T flocculation.

**FIG 4:**
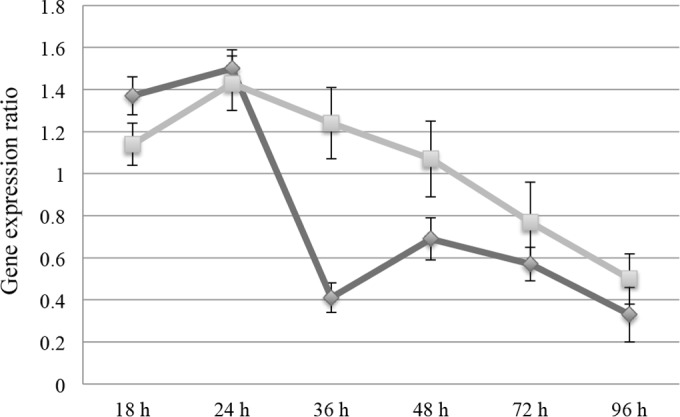
Gene expression ratios of EPS biosynthesis (diamonds) and EPS deacetylase (squares) normalized to GAPDH expression.

In order to determine how EPS biosynthesis (*tmz1t_3244*) and EPS deacetylase (*tmz1t_3249*) genes influence the differentiation of MZ1T from planktonic to floc-forming growth phases, we examined the gene expression profiles of the genes in wild-type MZ1T cultures over a time course spanning before and after flocculation using droplet digital PCR. The results (Fig. 4) demonstrated an increase in the expression of the EPS biosynthesis and deacetylase genes during the growth period from 18 to 24 h. At 36 h, EPS biosynthesis gene expression dramatically decreased and partially rebounded just prior to floc formation at 48 h and then decreased again throughout the full 96-h culture assay. In contrast, EPS deacetylase gene expression peaked at 24 h and gradually declined thereafter.

## DISCUSSION

Mutants of MZ1T that were reduced or deficient in flocculation were isolated following NTG chemical mutagenesis. Surprisingly, all the mutants produced some level of extractable EPS. Furthermore, analysis of the glycosyl composition of the EPS revealed the presence of the same basic monosaccharides found in the wild type. It therefore appeared likely that the mechanism(s) behind the loss of wild-type levels of floc formation among these mutants did not involve the basic production of EPS or its precursors. Instead, FTIR spectral data derived from EPS samples of flocdefective and reduced-floc mutants suggested that a modification of the EPS had taken place in the floc-defective mutants. Moreover, this modification was apparently conserved among all the floc-defective mutants but absent in the reduced-floc mutants 39A and 44B1. These data suggested a role of EPS modification in floc formation. The causes underlying the reduced-floc phenotype (e.g., 39A) might be explained if the EPSs produced by these strains were no longer associated with the cell surface or not modified to the same extent as in the wild type, but further work is necessary to clarify this issue.

**FIG 5:**
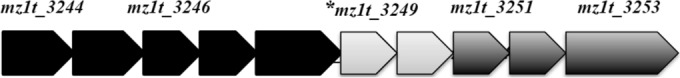
Physical map of the putative polysaccharide deacetylase genes located in the MZ1T EPS cluster (*mz1t_3244*, polysaccharide biosynthesis protein; *mz1t_3245*, hypothetical protein; *mz1t_3246*, lipopolysaccharide biosynthesis-like protein; *mz1t_3247*, asparagine synthase; *mz1t_3248*, family 2 glycosyltransferase; *mz1t_3249*, polysaccharide deacetylase; *mz1t_3250*, family 2 glycosyltransferase; *mz1t_3251*, hypothetical protein; *mz1t_3252*, family 2 glycosyltransferase; *mz1t_3253*, sulfatase [*wbbL*]).

Complementation of *mz1t_3249* (a putative polysaccharide deacetylase gene) in mutant 20A was found to rescue the flocculation phenotype. This gene is located in EPS cluster 1, believed to be involved in the biosynthesis and export of the extracellular polysaccharides likely required for flocculation in T. aminoaromatica MZ1T (Fig. 5). This EPS gene cluster has highly conserved gene organization compared to that of Rubrivivax gelatinosus. The latter photosynthetic bacterium changes from a planktonic lifestyle to a phototrophic biofilm when EmbRS, a two-component signaling system, is inactivated. Complementation of genes in the EPS cluster of *R*. gelatinosus also restored autoaggregation and fast sinking of the compact mass of cells in a Δ*embRS R*. gelatinosus mutant (13), confirming that this EPS gene cluster is responsible for biofilm formation and flocculation in R. gelatinosus.

FTIR results showed that the C=O peak of the acetyl group of *N*-acetylglucosamine and/or *N*-acetyl-fucosamine is reduced in the wild-type MZ1T EPS compared to that from mutant 20A, indicating modification by possible deacetylation in the wild type. This finding is consistent with a model of flocculation dependent on modification of EPS side chains and is further supported by complementation of the putative deacetylase gene in mutant 20A. However, deacetylation in the MZ1T wild type appears to occur only partially. Chemical deacetylation of MZ1T mutant EPSs using strong alkaline treatment and heat completely removes acetyl groups, whereas the untreated wild type and 39A mutants still have some degree of reduced C=O peak for the acetyl groups. It has been shown that partial deacetylation of poly-β-1,6-*N*-acetyl-d-glucosamine (PNAG), which is an intercellular adhesin, is crucial for secretion of the polymer in E. coli (14). In addition, the deacetylation of PNAG in Staphylococcus aureus and S. epidermidis introduces positive charges in the otherwise neutral PNAG molecule, as free amino groups are exposed that then become protonated at neutral and acidic pH. The cationic character of PNAG has been found to be essential for the attachment of PNAG to the negatively charged bacterial cell surface, leading to cell aggregation and biofilm formation (15).

Therefore, we postulate that the putative EPS deacetylase creates positive charges on the aminosugar glycosyl component of MZ1T EPS, which subsequently attracts the negatively charged carboxyl group of galacturonic acid, leading to interchain binding of MZ1T EPS and flocculation. The conclusion is supported by different colony-staining characteristics of MZ1T wild-type and mutant colonies described previously (10), where it was found that wild-type colonies could be stained with the hydrophobic dye Sudan black but repelled the acidic dye alcian blue. The results for floc-defective mutants were the opposite, indicating a more hydrophilic surface property, possibly increasing repulsive interactions and interrupting flocculation.

Interestingly, when expressed in E. coli, the putative deacetylase gene of MZ1T results in flocculation-like cell behavior in E. coli. The putative EPS deacetylase could increase the degree of deacetylation of PNAG, which in turn may interact with other polysaccharides found in E. coli. Further analysis will be required to define the mechanism, but the finding may be useful in biotechnological applications, where controlled flocculation would be a benefit.

In the MZ1T 39A mutant, due to the reduced floc formation in the strain, we speculate that direct or indirect regulatory genes may be involved in this defective phenotype. We found SNPs in genes for several response regulator receiver protein, PAS/PAC sensor signal transduction kinase, and diguanylate cyclase genes. It is well known that synthesis of the alginate polymer in P. aeruginosa is allosterically regulated by the secondary messenger cyclic-di-GMP (c-di-GMP), as binding of c-di-GMP to the inner membrane protein is essential for bacterial exopolysaccharide production, leading to autoaggregation and biofilm formation (16). In addition, the signal transduction cassettes ArlRS trigger and regulate biofilm formation of S. epidermidis in an *ica*-dependent manner (17). We also discovered a mutation causing an amino acid change in a glucose-1-phosphate thymidylyltransferase gene belonging to EPS cluster 1. Indeed, failure to synthesize an EPS precursor has been demonstrated to diminish the ability to form a biofilm (18). However, near-wild-type levels of EPS production by mutant 39A are inconsistent with low production of EPS precursors. Nevertheless, none of the candidate regulatory genes tested could restore floc formation to wild-type levels, suggesting that they are not directly responsible for the phenotype of the strain.

Expression of genes involved in EPS biosynthesis is often influenced by a diversity of environmental and physiological stimuli, including stress signals, nutrient availability, and temperature (19). Regulation of EPS production can occur at any level, such as transcription initiation, mRNA stability, and protein activity. In our study, expression of the EPS biosynthesis gene located in EPS cluster 1 increased during late exponential phase. It has been reported that in comparison with planktonic cells, recently surface-attached P. aeruginosa increases alginate gene expression (20). The expression of the MZ1T biosynthesis gene dramatically decreases between 24 and 36 h of growth and, surprisingly, increases again at 48 h, which corresponds to the time of flocculation. This phenomenon may involve posttranscriptional regulation, as has been seen in other systems (21). In addition, it is possible that a stress response signal(s) could participate in MZ1T flocculation, since increase of the EPS biosynthesis transcript occurs in stationary phase. Finally, the EPS deacetylase gene is constantly expressed before flocculation and is gradually reduced, indicating that the ratio of the gene to EPS-biosynthetic genes, which is greatest just prior to flocculation, may be an important driver of floc formation in T. aminoaromatica MZ1T.
